# Discussions at the Twenty-First Annual Meeting of the Mississippi Valley Dental Association

**Published:** 1865

**Authors:** Geo. J. Manson

**Affiliations:** Phonographer


### DISCUSSIONS

At the Twenty-first Annual Meeting of the Mississippi Valley Dental Association, held in Cincinnati Wednesday, Thursday and Friday, February 22d, 23d and 24th, 1865.

Reported by GEO. J. MANSON, Phonographer.

### *Anœsthetics.*

Wednesday, February 22d, 1865.

THE PRESIDENT: I confess to a continued long and cherished dislike to anæsthetics in any shape—either gas, chloroform or ether. I do not believe that the brain and nervous system can be acted on by any agents to preduce insensibility to pain in surgical operations without bad results following.

To be sure, extracting teeth causes pain, but the patient gets over it, and when chloroform is used, no one can tell how great an injury is inflicted on the lungs and nervous system. I would not give any of the anæsthetics for so simple an operation as the removal of a tooth. We know that several hundred fatal cases from the use of chloroform have occurred, and many of them in the hands of the best surgeons. We can not look through a man and know that his lungs and heart are in the proper condition to employ this powerful agent. There are two cases reported in which it was administered without apparently bad results, but when repeated some time subsequently for the removal of teeth by the same physician, and with as much caution as before, it caused the instant death of the patient.

Dr. Taft: This subject of anæsthesia is a very extensive, and a very important one. There are many things in reference to it that are too often left out of view. The paper read yesterday was a very good one, and yet it did not refer to some very important matters, in connection with this subject, that should be presented and discussed.

It is a well recognized fact that almost every person has peculiarities of his own. It is true in regard to the susceptibilities of various individuals to various therapeatic agents; it is true in regard to individuals with reference to articles of food. One person partakes of a madicine and it does him good, while it would do another harm. This fact is too little observed by us all. Now these peculiar idiosyncrasies we can not recognize so far as chloroform is concerned, nor so far as many other things are concerned. You know that some persons are easily influenced by chloride of mercury, while, again, others are not, and it is with great difficulty you can make an impression upon them. So with regard to anæsthetics—ether, nitrous oxide gas, and every thing of that kind; there is a difference of susceptibility in different persons.

We ought to study this subject. As yet, however, there is nothing or very little that is definitely known in regard to this susceptibility. Some persons will be affected by one of these agents in one way, while another person, apparently of the same temperament, in the same operation will be affected in an entirely different manner. Some persons seem to resist the influence of chloroform to a very great extent, while others, after a few inhalations become perfectly under its influence. I had a singular example of this kind a few months ago. A lady, perhaps thirty five years of age, called upon me to have some teeth extracted and desired to take chloroform. I refused, as my custom is not to give chloroform. She insisted, and so strongly, that I consented that she might bring her physician and have him administer it. She brought him, and as is now the approved mode, he administered a heavy dose of brandy before he administered the chloroform. He then gave the chloroform to her, and within five minutes she became entirely uncontrolable; she did not not loose her consciousness, but she was as wild and insane as a person could be. He administered chloroform to her for *fifty-five minutes* without producing the anæsthetic state. She was talking incoherently, and part of the time fighting some imaginary person. After administrating it fifty-five minutes she did not become insensible, did not become unconscious, because she knew every motion I would make, and would not permit me to approach her for the purpose of extracting the teeth. The physician who was there, was, probably, one of the best in this city, and he finally gave up, saying it was no use to administer the chloroform any longer, and that he would not press it. He was very cautions in the administration of it. As many as could get around the patient held her, when I took out five or six teeth, she struggling and making great demonstrations of suffering; after her recovery from the effects of it, she said she suffered. This was between ten and eleven o'clock in the morning. She lay in a wild disturbed condition, talking incoherently, until about five o'clock in the afternoon, not having her right mind, except occasionally in a lucid moment she would speak intelligently, about five o'clock she went home. In a few days she returned and desired the others removed by the use of chloroform. At first I refused, but, seeing that the physician had failed; at her earnest request, I consented to give the chloroform myself, and thought I would press it a little stronger than the physician had done. I gave her a portion of brandy as before, and then administered the chloroform, pressing it as closely as I dared to do, keeping the cone, part of the time, directly on the the mouth and nostrils; I administered the chloroform for an hour, letting as little atmosphere as I dared pass into the lungs. Three minutes after she began to inhale, the same condition came on as before, and while she was in that wild state, I took out two teeth; she was so unmanageable that it was impossible to take out more. She remained in that condition at the office until ten o'clock that night when she was taken home in a carriage. She continued in that state for two days. She slept little during that night. How it is that many of these cases fall on some few unluckly wights, I can not say. This was a very peculiar case. She had all the advantages that she could have; she took the stimulant in the first place, it being administered, as is the custom with almost all surgeons, she took it in ample proportion at both times. The first time the chloroform was administered very cautiously and an abundance of atmosphere permitted to pass into the lungs. The second time it was pressed as closely as was thought admissible, giving only enough atmosphere to retain life. The last time the condition lasted longer because the chloroform was given stronger. There seemed to be a state of hysteria that, previous to this, was dormant, but was aroused by the agent, and made its manifestation in the manner I have described. I only removed two teeth then. She came back in a few days afterward, when I said I would never administer chloroform again to her, and she should never take it if I could prevent her, and she *must* let me take out those teeth. After a few minutes she quietly sat down and I toook out fourteen teeth without her struggling, and scarcely making a contortion of the countenance. A little firmness and decision by an operator is often a great deal better than chloroform. You can many times do every thing that is necessary if you simply have firmness and the influence—the magnetic influence—over a patient, and that every person can have if he will. It is only these hesitating, fearful, timorous persons that would fail to have that influence over their patients. If an operator can not have that kind of magnetic influence over any and every patient, it would be better that he did not operate for them, and that he simply sent them away saying, "I am not the person to operate for you; get some one else." I believe we operate for a great many persons we ought not to touch. There is many a time with me, I know, a feeling of repulsion between my patient and myself. I feel as though I did not want to be near him. I presume that all dentists occasionally have that feeling. There are some patients with whom you love to talk; for whom you love to work; when you operate for such an one you do not weary, though the operation might be a protracted one. With such patients you will do the very best you can; while, if you work for one between whom and yourself there is this feeling of repulsion, you can not produce the best results.

I have no doubt but that there is a large amount of injury inflicted on persons by the administration of chloroform—injury that drags along and exists for a long time—and, perhaps, puts them in a condition from which they never recover. How far we may injure a person in this respect, we do not know, but we ought to study these things.

There is another objection to the use of this agent, viz.: its influence over the operator himself. If I knew that it would not injure the patient at all, and had its present influence over the operator, I would not use it as a practice. I

am not willing to injure my own health, and dement myself in any operation whatever.

In regard to the idiosyncrasy or susceptibility of patients to chloroform, we know little or nothing . Patients that have been regarded as the best subjects—and by persons who administered chloroform for many years—have died from its influence. I think SNOW administered it in ten thousand cases without loosing one, and then had after that a number of fatal cases. He studied idiosyncrasy, but he failed to rccognize the unfavorable subjects—failed after his long experience. Three of the best surgeons in this city lost a case the other day—a man whom they supposed to be a good subject. They administered chloroform for a simple operation, and he died on their hands. They did not recognize any peculiar idiosyncrasy, and they are men at the top of their profession, and who have administered chloroform in perhaps thousands of instances.

It seems to me that dentists should be exceedingly cautious in this matter. And yet what will your caution amount to if you do not know what this liability to accident depends upon? Death occurs in two ways by the administration of chloroform; first, by its specific action on the nerve centers, then death is instantaneous; the heart's action is stopped instantly, and the motion of the respiratory organs ceases. The second way is by asphyxia—by the suspension of the action of the respiratory muscles, and of the action of the lungs, and a relaxation of the glottis.

We can not understand the cases in which these peculiarities and susceptibilities exist, any more than we can understand the peculiarities that make persons susceptible to any other medicinal agent. Can you say to a person, "You are a good patient for the administration of mercury in any form?" Can you make any such distinction definitely? Have *we* got far enough in medical science to recognize these idiosyncrasies? We have not. Medical men claim no such attainments as yet.

A word in regard to the ability a person should have in administering chloroform. In the first place he should understand the anatomical, physiological and pathological condition as perfect as possible. If he does not, he is going in the dark. But a person may have all this knowledge and be perfectly incompetent to administer chloroform.

Another thing is necessary, viz.: that the person administering it shall have perfect reliance in his own ability. He must have the power, and the ability to do the very best thing in the greatest emergency that may come upon him. There is great difference in these cases. An occasion may come when he may be unnerved, his power gone, and when he may stand back in horror from the patient. We had example of that in our profession recently. A patient died in the chair of a dentist. The instant the dentist saw there was a difficulty arising he left the room and ran out a few squares for a physician. How much reliance had that man in his own ability? The man who attempts to administer chloroform must feel that he has the ability to do the best thing on a great occasion. Occasionally you find a man who, in a great emergency, has more power brought out than he supposed was in him. But the rule is, that, when a great emergency comes, we are unnerved; we do not know what to do; we forget every thing we knew before; such a man should not administer chloroform, whether he is physician, surgeon or dentist.

I do not believe its use is admissable at all, unless in extreme cases. I would make this as a rule, that where there is less danger from the operation than from the administration of the anæsthetic, I would not use it. I do not believe that there is one case in a thousand where it is proper for the dentist to administer chloroform or ether for the extraction of teeth.

The PRESIDENT: How about gas?

DR. TAFT: In regard to gas, I consider it a safer anæsthetic than chloroform. It produces the condition in a different way. I regard it as more admissable than chloroform, and I think better than ether. One respect in which it is better than ether is, that it acts more promptly, and there is not the disagreeable results from its administration as from ether. A patient put under the influence of nitrous oxide gas passes from it without any unpleasant symptoms; that is not true of ether. There is a great objection to the present manner of the administration of nitrous oxide; I refer to the time and apparatus employed. It requires time that we can not afford to spare in ordinary practice. I regard it as the best anæsthetic there is. But there are many cases in which even that will not be successful. There are cases where patients have said, after the use of the anæsthetics, that they suffered as much as they would have suffered had they not taken the anæsthetic. The great majority of persons, of course, can be completely brought under the influence of chloroform and made entirely insensible. But then, even these persons will sometimes make demonstrations of pain.

[The election of officers for the ensuing year was then proceeded with; the result is given in the minutes of the meeting, published in this number.]

Dr. CHEESBROUGH, the new President, on taking the chair, addressed the association as follows :—

*Gentlemen of the Association :*—I can not but say that I thank you for the honor you have conferred upon me, to be your presiding officer for the coming year. We meet under circumstances of no small moment. Our deliberations will be transmitted to others, as well as be listened to by those who are present. Let us, therefore, in our deliberations try and render them so, that we shall not be ashamed of any record that may be made of them hereafter.

I can say from my heart that I think it an honor to preside over an association comprising, as I see before me, so much real, thorough, true worth. I think, from past experience, that the Mississippi Valley Association has no equal.

Of the members composing it, I consider that for intelligence, for honor, for integrity, for everything that makes the *true* Dentist,—you can look over all sections of the country, and not find so many gathered into any one association, (if I may except one,) of all the societies whereof we are acquainted.

Let us strive to do our utmost. Let us look deep into everything that we are called upon to discuss. We had last night, from the mouth of Dr. Taft, the advice to the graduates: "Read some—write much—investigate a great deal." May that be the motto of this Association, and let us bring it up to the standard that may be required of any institution of its character.

Gentlemen, I thank you again for the honor conferred upon me.

Dr. McClellan,—A few words upon anæsthetics. I am not in favor of them as a general thing. The more experience I have with them, the more cautious I become. I do, however, occasionally use them. The rule Dr. Taft laid down is a very good one if we could always apply it,—whether the patient would suffer more to have the operation performed without the anæsthetic than he would with it, is a point pretty difficult to decide, and requires a man of considerable experience in practical cases to pass upon it.

There is in the use of all anæsthetics a period of excitement. That was the period which Dr. Taft made allusion to in the case which he detailed to us. That case was not carried beyond the period of excitement. Had that patient been mine, I should have taken the responsibility of pushing the anæsthetic effect beyond that point. I do not think there is as much danger from the use of anæsthetics where this excitement is manifested, as where it is not. I am always more cautious in administering an anæsthetic to a patient where no excitement appears, than where it seems to have a depressing effect.

I proceed very cautiously in the latter cases, and would recommend the administration of a stimulant, more particularly in cases like that. Even after I have commenced to administer the chloroform, if I see it is going to have a depressing influence, I cease its administration and give a little brandy or whisky.

We are often applied to, to administer chloroform without knowing anything about the constitution of the person; we need experience to gain knowledge of the constitution from appearance.

There are some few cases where I would recommend its administration. In cases of children, where they are suffering from inflamed gums, alveolar abscess, and where they are very excitable; I would not hesitate to administer chloroform, thinking there would be less injury done by administering the agent than by the operation being performed without it. There was a case published a few days ago, of death from removing teeth by force from a child in the City of New York. I presume it was a case of this kind, where the child was very excitable, and they held the child to extract the teeth, and death was the result from fright.

I never administer chloroform to stout healthy men. In war times, if they have not courage enough to sit down and have their teeth extracted, they had better advertise for a substitute. (Laughter.) There are some ladies to whom I would advise the administration of chloroform. Those very nervous, excitable patients sometimes, are very favorable cases for chloroform, if they are not afraid of the agent they are taking. If they have much fear on that score, I do not give it; but if they say they have taken it before, and insist on it, and if I am satisfied that they have no well seated organic disease, I do not hesitate to give them chloroform.

A few remarks on the treatment of patients while under the influence of chloroform. In the first place, it is requisite that every thing be quiet; that the patient should be placed in a comfortable position before he takes the chloroform, so that it will not be necessary to adjust the body or extremities. To illustrate this: I had a case recently, of a young lady who took chloroform, and seemed to go under its influence, after a few inhalations; just as the muscles relaxed, her foot slipped off the foot-stool, which startled her; she roused up and said, "Don't take out my tooth yet," though she was evidently acting under the influence. After that it was almost impossible to bring her under the influence again, although I administered it to her twenty minutes; but she finally yielded.

I have seen chloroform and ether administered at the same time. Two ounces of ether was administered first, and then four ounces of chloroform. This was by one of our most experienced physicians, who has had a great deal of experience in obstetrics. The patient came under its influence, the teeth were taken out, but was quite drowsy, and was allowed to lie down on the sofa and take a nap, being placed in such a position that the blood might be drained from the mouth.

It is well to have plenty of napkins on hand, and if the gums bleed freely, to wipe out the mouth and keep it dry. Keep the fingers as free from blood as possible, as if friends are by, it makes the most careful operator feel sensitive to have them see it.

A few words about nitrous oxide. Having had some experience with that agent, I would say it has some things to recommend it. One very strong recommendation to it is, the feeling we have as to its being a less dangerous agent than chloroform. I must say I have administered nitrous oxide to persons just as they have come in. I have used it for some months. There have been published some cases of death from using it, but they were not well founded, and it was afterwards ascertained that the patients died from other causes. So far as I know, I think there is no well founded case of death from the use of nitrous oxide when administered for extracting teeth. The best method of preparing it, as I am instructed is, from the salts of ammonia; it should be very carefully made, not evaporated too rapidly, and passed through a large body of water.

I believe any one can be brought under the influence of the nitrous oxide. I have seen the stoutest men brought under its influence in less than one minute; and within three minutes the teeth were out, and they were perfectly conscious, and feeling as well as ever.

I have had patients who, when they took it, complained of headache, but when they left my office they said they felt better than when they came in. I have never failed to bring any one under its influence when I had fresh gas; and have taken out as many as six teeth under one administration of nitrous oxide. I had one case where I could have taken out all the teeth of the patient, before he revived, he being in a calm sleep. I only have had one case where I felt any alarm, and that was an epileptic patient. A very pale looking subject, a lady, came into my office one day, and desired an anæsthetic, as she wanted some teeth extracted. She desired to take nitrous oxide gas; after trying to induce her not to take it, I agreed to administer it to her. I did not hesitate to give her the gas, not knowing the tendency of the disease. A lady friend came with her,—gave her the gas, and took out the tooth. When she revived, she immediately went into an epileptic spasm. That did not alarm me much, as I had several cases of that kind before. The lady with her was very much alarmed. As soon as she revived sufficiently to speak, she called for salt and water, and then went off into another spasm. By the time she revived again, we had the salt and water prepared, and she eagerly drank down half a tumbler full. The first word she said when she revived was, "Doctor, don't think it was taking this gas that caused it; I have often had these fits." That was the only case I ever had where any thing unpleasant occurred from the gas.

One recommendation of nitrous oxide is the rapidity with which it acts. It is not so good in prolonged operations as chloroform, but if you have one or two teeth to extract, it is *the* thing for the Dentist. A tooth or two can be taken out by this means within three minutes.

Some of us do not like to give anæsthetics. There is a way of refusing to give them without acknowledging we are afraid to. I charge extra for administering the gas, and an extra fee for allowing it to be administered in my office. Since I have adopted this, I have not near so many applications to give it as before. I charge $5 00 extra for administering the gas, and the same price for allowing it to be administered in my office; from $5 00 to $10 00 for chloroform, and $5 00 where it is administered in my office. My reason for doing this is to prevent so many applications. Sometimes patients consume much of an operator's time uselessly.

Dr. Dean; I wish to say a word on operating on the patient while he is under the effect of anæsthetics.

I had a young man come into my office, of a very nervous temperament; he had some seven or eight roots to be extracted. He desired to take chloroform; had tried the gas, but he was one of the nervous kind and got his mind made up that he was going to laugh, and so he did laugh, and was so boisterous that nothing could be done with him. Knowing that peculiarity in his disposition, I supposed he would act the same while under the influence of chloroform. I knew it would be very hard work, under such circumstances, to get his mouth open—perhaps you have all had difficulty in that respect. I had every thing perfectly quiet in the operating room, and kept saying to him, "Keep the mouth open; let that be the only thing you are thinking of; keep your mouth open as wide as you can." And he did keep it open; it was perfectly astonishing with what facility I extracted the teeth. When he went under the influence of the gas, his mouth was wide open, but he shut it soon; and when I was ready to extract the teeth, I asked him to open his mouth, which he did. When he was coming from under the influence, and after I had extracted the teeth, so firmly was that idea fixed in his mind, that he said, "Is my mouth open wide enough." I have since adopted the plan, telling the patients to keep the mouth open, as I did him, and I have not failed in a single instance to make them obey.

Dr. BERRY: There is a member of this Association, who is not present, who knows of a very interesting case of the effects of nitrous oxide. It was administered to a student in a literary seminary, with which this gentleman was connected, by a good chemist, who knew as well as any one how to administer it. The student was a very talented young man, and the result was (it occurred quite a number of years ago), that he was made so nearly an idiot as to be unfitted for usefulness in after life.

Dr. ULREY: I do not want to speak on anæsthesia; I have never tried nitrous oxide gas,—I used chloroform years ago. I desire to have both sides of the subject considered, for the benefit of the young men who are present and have not had much experience in the management of cases. I had some considerable experience in using chloroform, but have had no accident occur from its use.

The PRESIDENT: I have given a good deal of attention to the administration of nitrous oxide gas as an anæsthetic; I have administered it a great deal. Dr. Berry states a case where a derangement of the brain transpired from the use of nitrous oxide gas. I am glad he stated that case; that, however, is no reason why that agent may not be given to mitigate pain. We have seen persons who, from over eating, or under eating, have been injured; but is that any reason why we should not eat? I am not a strong advocate of anæsthetics by any means, and whenever I can get along without administering them, I do so; I think it is better that the patient should come right up to the mark and have the work done; then there is no danger of any after-clap. But there are some persons who have such an unmitigated dread of pain, that it is impossible for them, for one minute even, to think of it without going into a paroxysm. In such a case as that what is our duty? If we have an agent that we know in the majority of cases succeeds—never fails, say once in a hundred thousand times, our duty is to use it. We know that chloroform and ether are given a great deal in surgical operations, and it is well that they are. I doubt very much whether the Doctor (Berry) himself would find so much objection to anæsthetics if he had a çapital operation to be performed—something that would bring him under the exquisite torture of the knife; I think he, himself, would be glad to have the pain mitigated, and would even come down to nitrous oxide. I know the Doctor's dislike of some things is strong; he has his prejudices; so have we all. It may be but a difference of opinion, and we are told that every difference of opinion is not one of principle.

In regard to the administration of chloroform and ether, I did not dwell so long upon that subject in the paper I read to you yesterday, as I should have done, had I had more time. I can not press upon your minds too strongly the importance of having on hand, at the time of the administration of either chloroform or ether, nitrous oxide gas as a resuscitating agent. I would never give chloroform or ether unless I had that material by me so that I could apply it to the patient and resuscitate him. Dr. Taft has given us an account of the case of a patient who died in one of the hospitals from taking chloroform for the simple operation of fistula in ano. I doubt much if that patient would have died had nitrous oxide been given him when the physicians in attendance saw that he was sinking. In my own practice I have given it three or four times, and found it to be of the greatest benefit. I have seen reports of cases where nitrous oxide gas has been given with a view to resuscitate life. I have seen reports, I think, of twenty-eight cases, where it was administered to the soldiers in the hospitals at Nashville,—soldiers who were, by the physicians in charge, given up as in the last stages of life. I think of that number twenty-three recovered, and are now healthy men. This was at a time when anæsthetics were not much given for the extracting of teeth, and I think the physician who advised the administration of it was a Hungarian, and we see how his efforts were rewarded. Suppose he had saved only one life out of that number, would it not have been a sufficient guarantee that had he administered it to the others they would not have died. In cases where we can administer a substance to a patient without fear of incurring death, I certainly think that we are warranted in doing it.

Dr. Berry: The President misunderstood my remarks this morning on the subject of the use of anæsthetics. I censured the use of chloroform for operations in minor surgery. I do not stand here to censure the whole surgical world for using it in severe operations. I do not suppose we have a well authenticated case from the time of the father of medicine to the present, where death resulted from the extraction of teeth. That being the case, we are not justified in running the risk of life and health in administering gas, chloroform or ether.

Dr. Taylor: At the close of the reading of Dr. Cheesebrough's essay yesterday, I intended to make a few remarks, which I will make now, but they are not in the order in which I should like them to be.

The first point is: the cases which nitrous oxide must fail to restore. The Doctor remarked that after the administration of chloroform or ether, the chlorine acted so upon the glottis, at times, as to produce a spasm and stop breathing. I had two cases in my office which illustrates that remark. When breathing has suspended I have found that no agent whatever applied, has any effect at all, so far as restoration is concerned on the breathing apparatus. The first thing we must do is to restore the glottis to its natural function. If you had a patient prostrated, whose pulse was still beating, (and I have had such cases,) your nitrous oxide fails—fails because it is not inhaled. We all admit that oxygen is the life principle—so if we could get it to its place it would be the agent we want. Perhaps, if we could get the gas into the lungs it would be the agent, but I have found that there is enough oxygen in the atmosphere, when we can get the lungs to play, to restore the patient. I have never failed when I could get the patient to breathe. I recollect a case of this kind. I administered chloric ether until my patient became insensible; I took out three teeth, finishing the operation in one minute. I then noticed a peculiar look in his face; the pulse was feeble, but still circulation was going on. I threw some water in his face, and put the ammonia bottle to his nose, but there was no breathing. I lifted the patient forward, shook him violently, and intended to put my finger in the mouth and remove the difficulty, but by bringing him forward and violently shaking him, the difficulty was instantly removed, because the air passed into the lungs.

There is another point I will touch upon. It was stated in the essay that "we can not give ether where there is any difficulty about the lungs." I have administered chloroform and ether both, where there was organic lesion of the heart, and of the lungs. Take the fatal cases occurring in CINCINNATI; not one has resulted from any difficulty of this kind. They appeared to be fit subjects for these agents. The first woman who died in Cincinnati from the use of anæsthetics was a stout, athletic woman, whom nobody would have thought of dying. A large majority of the fatal cases have not been where there was disease of the lungs or heart, but where there was generally plethora. If a patient has organic lesion of the lungs, and asthmatic difficulty, and you give him ether, it diminishes rather than increases the difficulty of breathing.

### *Cases Where I Would Use Anæsthetics.*

I would use an anæsthetic for the extraction of teeth just as a general surgeon would for a capital operation. If a patient comes in for the removal of one or two teeth, I do not use it, but if from eighteen to thirty have to be extracted, and I find there will be less danger in using it than if I did not, I use it. These are the only cases where I would use the agent. There are two or three reasons why I do not always use it. In minor operations, we should educate our patients up to that point where they will endure anything of this kind. I have less trouble now than formerly. A patient asks me, "Do you give ether or chloroform?" I answer "no," and as a general thing they sit down and have the teeth extracted. I do this on the ground that the risk is greater than the benefit. There is only one way—you must either put your price beyond the range of common persons, or get rid of the operation entirely; I prefer the latter. When I give ether or any such agent, to my patient in the morning, I am generally unfit for work for three or four hours of the day. My experience has been such that, for the last year, I have only administered it two or three times. Some two years ago, after a severe days work, I concluded to give ether to a patient for the removal of some twenty teeth, and the result was that I was not able to pull a tooth for a week.

### *The Best Mode of Administering Ether.*

My partner administered ether recently by putting a handkerchief over the patients face, but previously some oil silk to prevent the ether coming in contact with the face; we make a funnel and administer it, in that way a portion of it is not inhaled and the patient breathes in the atmosphere and it takes one quarter more to bring him under its influence than it would otherwise.

Dr. H. A. Smith: I regard chloroform or ether as the best and safest anæsthetic now in use. Of these two I prefer chloroform, as its effects are much more rapid and the patient more easily controlled than when ether is administered.

In the essay read yesterday, it was stated that alcohol deprived of water produced the same effect that ether does. This I thought was doubtful; though it is true that alcohol deprived of its water has the same constituents in the same equivalent proportions that ether has; yet alcohol can not be converted into ether by taking from it the water.

The use of these anæsthetics is always attended with more or less danger to the patient; and I thought they should not as a rule be administered when only one or two teeth are to be extracted. If several are to be taken out, it is equivalent perhaps to a capital operation in surgery, and there is more reason that anæsthetics should be given. No doubt these agents are administered in hundreds of cases, when, if the dentist had taken a decided position that they should not be used, the patient could and would have submitted without much reluctance to the operation of extraction. The same person should never give the anæsthetic and extract teeth also. In the best conducted hospitals a chloroformer is employed. To extract teeth successfully requires the whole application of the mind of the operator, and he can not at the same time give the attention to the patient that is necessary to observe the earliest symptoms of danger. It is only by attention to the first signs of danger that the life of the patient can be saved.

It is a greater risk than I am willing to incur to administer an anæsthetic and operate at the same time.

It was stated in the paper that carbon would produce anæsthesia. I do not think so; carbonic acid gas might, but I doubt it.

The President: I think the gentleman misunderstood me. I said that ether, chloroform and alcohol were each composed of certain elements; that alcohol has for one of its constituents one equivalent of water; if that water is, by any means taken away from it, it becomes ether; that equivalent of water is taken out of it in the manufacture by the use of sulphuric acid, the product being sulphuric ether. I made no assertion to the effect that if "alcohol was deprived of its one equivalent of water it would be chloroform;" it was ether to which I alluded.

Dr. H. A. Smith: I did not misunderstand you. I meant ether. If you take from alcohol the water, although it possesses the same constituents as ether, I do not think it will produce the same effects.

The President: I think the chemical authorities say it will do so.

Dr. H. A. Smith: It looks as if it would, but when tested I find that it does not.

Dr. Taft: I think all who administer chloroform, recognize the fact that its influence is much modified by the condition of the digestive apparatus.

Dr. Baxter: I have not had a great deal of experience in the use of anæsthetics, but I have occasionally to resort to them. Before administering I always examine the stomach. I never administer it on a full or empty stomach, nor when the bowels are distended. As to anæsthetics for extracting the teeth, I oppose all of them for the reason that they have a deleterious effect upon the patient.

Dr. Taft: I like the suggestion Dr. Smith made in regard to having the administration of chloroform put into the hands of one person. In a city like this, there might be a person who would attend to the whole of that business, for all those who are disposed to use it; I think in every locality where chloroform is given much, something of that kind should be instituted. This plan would apply just as well to the surgeon as the dentist. This, to a large extent, would be a shield to the operator, and would be quite practicable.

Dr. H. A. Smith: The custom of calling in the "family physician" to administer chloroform to our patients, is a good one, provided he is familiar with the administration of anæsthetics. If the physician is one of that timid hesitating kind sometimes brought to our offices for this purpose, we should in justice to ourselves either dismiss the case or assume the entire responsibility ourselves, as indeed we would have to if he were allowed to remain. If the chloroformer exhibits any timidity when it is once decided to give an anæsthetic, the patient partakes more or less of the same feeling, and the effects are not nearly so certain.

Dr. Horton: As some gentlemen have given their experience on this subject, I should like to give mine. I am in favor of administering anæsthetics, and there are many patients with whom it is impossible to do anything unless anæsthetics be administered. Every dentist here has performed operations of this kind for some of the rising generation; even children from two to three years old have such operations performed; such cases occur very frequently with me in Northern Ohio, where what are termed the best families indulge their children. Every one extracting a tooth for a child two and a half years old knows, when they are in their right mind, he has more than he can do. The case which has been mentioned as occurring in New York of the death of a child, resulted, I think, more from some internal rupture than from the operation of extracting the tooth. In the cases of children I always have one of their parents present (generally the mother) when I saturate the napkin with chloroform and let the mother hold it from four to six minutes over the mouth, the child inhaling it; the child is soon in a sleep, when I take the napkin myself and administer enough chloroform to produce the desired effect. I have administered chloroform for the last thirteen or fourteen years, and sometimes as many as six times a day.

There are other cases where I would administer it. I mean in cases of females where they are in a peculiar situation and have gone, perhaps, to the seventh month; where they are suffering intensely from pain in the face, from alveolar abscess, or neuralgia, resulting from that, and where they are afraid to have the teeth extracted but not afraid to take the chloroform. I have administered it in many such instances and with perfect success.

In regard to the condition of the stomach, I consider that the most important item as warranting success in the administration of chloroform. There are two or three points which I always observe: I would never give it to a lady without having some friend of the family present, although I have an assistant. If they come to me within an hour of an ordinary meal I refuse to give it. I usually give it in from three to five hours after an ordinary meal; perhaps in not one case in a hundred will it then produce nausea. Where I administered it within an hour, or an hour and a half of a meal, nausea ensued once in about every fifty cases.

As to nitrous oxide I have had no experience with it, as the apparatus which was brought to me was so large and bulky that I refused to have anything to do with it. When it is used sufficiently to be known as a success, and the apparatus improved, I think I may then administer it.

As far as ether is concerned, in a good many instances I have had to mix chloroform with it. I think, on the whole, it would be better to mix it in the proportion mentioned in the essay read yesterday by Dr. Cheesebrough.

I never had but one fatal case, and I do not think that resulted from the use of chloroform.

I will state it: About two years ago this month, February, a lady, a stranger to me, came into my office and said she wished to take chloroform to have several teeth extracted. She came in about twelve or half past twelve o'clock on a Tuesday, and I administered the chloroform in the presence of a lady friend of her's. She inhaled the chloroform in the ordinary way, and I extracted three teeth for her. She recovered and was in the office perhaps two hours after. It was rather a stormy afternoon, and she staid till about four o'clock; in the mean time had perfectly recovered as I supposed, walked about the office, talked with her lady friend in a manner that induced me to think she was entirely over the effects of it. About half-past four o'clock she started to go home, went down stairs, forget her rubbers and came back for them saying, also, she wished to borrow an umbrella. (I mention these facts to show her state of mind.) She walked home, about a mile and a half. The next day she attended to some baking and other things. At noon on Wednesday a young woman came in to see her and talked to her in relation to the chloroform. In the afternoon, perhaps two o'clock, another woman came in and inquired how she felt after taking the chloroform. She said "pretty well, but sometimes she felt nauseated."

She had had that feeling previously. The lady said to her, "Perhaps you had better have a cup of coffee; I think that will do you good," and immediately went about preparing some. She drank a cup of coffee about three o'clock, and about four o'clock was attacked with severe spasms and vomiting. They continued till the next afternoon; at times she was deranged. About four or five o'clock on Thursday afternoon the young woman I mentioned (who had taken dinner with her on Wednesday) called in to see her, and then stated to her what she thought to be the trouble. She said there had been some family difficulties between herself and the woman who gave her the coffee; this woman had sworn vengeance on her and she supposed she intended to poison her, and as she had just taken the chloroform it would be a good time to do it, as the fault, if any, could be laid to that. There were several suspicious circumstances about it. In the first place, she had not felt unusually neauseated until after this woman had administered the coffee to her. Immediately after giving her the coffee she emptied the coffee pot, cleaned it and put it away. The pationt lived until the next afternoon about four or five o'clock when she died. A *post-mortem* examination was held by one or two surgeons, one the family physician of the woman who administered the coffee, who asserted positively that it was the chloroform that killed the woman; no other physician concurred in the statement, though many were summoned. I had an intelligent witness as to the influence the chloroform had on the patient, and her conduct while in my office, and on her return home. I have no more idea that chloroform killed the woman than I have that a person who falls in the water, is subsequently resuscitated, and dies of something else, is killed by drowning.

I have administered it in cases where I knew there were pulmonary difficulties. I should be loth to administer it in a case where there was any heart disease or any organic trouble of the heart in any way or shape.

Adjourned till three o'clock P. M.

### AFTERNOON SESSION.

SUBJECT—*Filling Teeth: the Material and Instruments Used.*

DR. TAFT: Mr. President—Many think that we have reached about the ultimatum of our knowledge on the subject of filling teeth, but there are continually being introduced little things to facilitate our operation in this branch of practice, and we hardly know where the stopping point will be. Some think there is no great improvement being made, but I think, in many points, there is. There is a constant effort being made to improve the method of operating. This is evidence that perfection is not attained; that is, there is something else desired, and some improvement sought for. There is a constant and rapid change taking place in the profession in regard to filling teeth; witness the use of the mallet, a very important change, and one that will altogether change the character of operations. I know the character of my operations is all the time improving. So it is, one thing after another is being introduced to improve the character of operations and more effectually save the teeth. This improvement consists in a great variety of little things, we see these every day without thinking much about them. For instance: Dr. McClellan has here in his possession a little foil crumpler. I believe it was suggested some time ago, but it was not brought to the notice of the profession in a tangible form until just now; it is an instrument that facilitates one part of the operation of filling teeth, viz.: the preparation of the gold for introduction. You perform that part of the work quicker and much better, by the use of that instrument, than by the ordinary method of crumpling in the fingers, and then, too, the gold is free from the moisture of the hands.

Then, again, there are other little things. The doctor has a little cap or a dam to put around a tooth to exclude the saliva. That is a little thing considered in itself, but it is an important thing when it will serve the object of excluding the saliva from the tooth when you are filling. Not long since some one showed me something similar to that: a small steel spring that may be wrapped with paper or cloth and clasp around the tooth, excluding the saliva.

There are other things besides these, small in themselves, but large in the results attained. When I look at the present method of manipulating by dentists; when I look at the manner of preparing cavities, at the excavaters, at the pluggers, and all the appliances used in filling teeth, I can not but think that our operations are far from being perfect, that they are scarcely ever made as good as they should be. A saving of time and labor in any of these operations, is important, and anything that tends to improve in these directions should be hailed by us with pleasure. I am always glad to see these little things, because there is something good in almost every one of them.

We have a great many in the profession who will not test new things. Many a man says, "Oh, you're not going to catch me with that; I will let somebody else try it, and after they have been at the trouble and expense of trying it, if it works well, then I will take hold." It is the *duty* of every man to try everything that promises any good. You will find, even after others have tested these things, that you must try them yourself. An appliance that works well in the hands of one man, in the hands of another will not work at all. It is important that every man should test for himself. I do dislike to see a man say he will let somebody else try an improvement, and, when he finds it will work all right, come in and reap the benefit of his experience. This is a wrong spirit. To perform our work we should take right hold, and do what lies in our power, and not wait for somebody else to open the right road for us. Every man has his part to do, and should do it.

Dr. Shadoan: I do not know that I have anything new to offer on the subject of filling teeth, but I will state my method. I have found long since that I can not use many kinds of material for the same purpose and do each equally well. I consider that gold is the best material for filling teeth. I use adhesive gold No. 4. Some, however, think adhesive gold is not so good as soft foil. In proximal cavities of inferior molar teeth, we can not use it to such advantage as soft foil. I now use adhesive foil almost altogether. One reason is, if we use that at all we must use it altogether, or else we can not manipulate so well in soft foil when we wish to use it. It does not require the same effort to manipulate adhesive foil as it does soft; when we train the hand, therefore, to the use of adhesive foil, the same training will not apply to soft, consequently if we change, it will reduce the standard of our operations. If we get in the habit of using No. 6, and then change suddenly to No. 4, we can not manipulate so well. I claim we can not use soft and adhesive foil alternately, and bring our operations to the same standard as if we used one kind alone. I regard No. 4 adhesive foil as the best material for filling teeth, because if it is properly manipulated, a filling can be made as hard at the bottom as it is at the surface. We can build against a thinner wall with the adhesive foil better than with the soft, and can manipulate quite as readily. If we have two very thin incisor teeth, we can not do as well with the soft as with the adhesive foil, because it requires too much pressure upon the tooth, and the strength of it will not permit of the requisite amount of force to use soft foil. Some cut away more of the tooth than others; I am in the habit of cutting away until I get a sufficiently firm wall to work against. If the edges are thin and ragged I do not stop, but keep cutting, unless it is in the front teeth, then I would leave a little surface that I would, under other circumstances, remove.

The next best material for filling teeth, in my opinion, is "Wood's Fusible Alloy." I have been using that material for about three years. I was a good deal like that class of whom Dr. Taft spoke, I did not care to use the material at that time, because I did not know whether it would suit. Dr. Taft spoke to me about it, I tried it, and have filled a great many teeth with it. I have taken out plugs that have been in from one to eighteen months, and have never yet found an instance where the cavity was in an altered condition. I never used it in filling over an exposed nerve. If the nerve is diseased, I destroy it, and fill the pulp cavity with gold first, and then fusible metal afterwards.

A Member: On taking the plug out at any time did you find it stained yellow?

Dr. Shadoan: I never have; I think if the plug did not fit close in the cavity, the secretions would cause it to turn yellow. I have heard other dentists say that they have found this yellow deposit inside the cavity, but I think this is more from not filling close to the walls of the cavity than any thing else; the fluids of the mouth having a chance to get inside the cavity. I have filled some very shallow cavities with the metal, and find it is sufficiently tough not to break or tumble out, and sets perfectly firm. In a great many teeth where the crown is pretty much gone but a part remains, I have restored the crowns to nearly their shape and size. I restored the crown of a tooth for a boy last spring, and should think if the plug had been made from gold No. 4, it would have required from forty to fifty grains. I saw the case the other day; the filling was just as good as when put in, and I am confident no other filling would do so well. Another reason why I prefer the alloy is, that it does not discolor the teeth, even if you place it close to the enamel.

Dr. METCALF: In connection with this subject of filling teeth, perhaps it is a proper time for me to exhibit a little apparatus that I have. It is pretty generally understood that dentists are using adhesive gold foil in one form or another. [Here the Doctor exhibited his lamp for annealing gold foil.]

In annealing adhesive gold foil, I think it is better to use this lamp, as the gold is not affected by the moisture of the breath. Abbey's foil requires to be heated red hot; White's only requires a little heat.

Dr. KEELEY: I have annealed foil by this lamp, and I never had gold work better; from the experience I have had with the apparatus, I have been very favorably impressed with it.

Dr. DEAN: I have used this apparatus, and another one on the same principle, and like them very much. It works very nicely, and as Dr. Metcalf says, it obviates the difficulty of having moisture as when annealed by hand.

I wish to say a word on filling teeth. Dr. Taft spoke of consolidation by the present system; perhaps you know what I am driving at; it is—*mallet.* I have not got "mallet on the brain," because I have not used it long enough. I like it, and have used it about two years, and have been trying to get up a method to obviate the necessity of having an assistant. I have got up a little instrument to take the place of the mallet, called a spring plugger; and it works as Dr. Taft said Hayes' most improved Vulcanizer works, *i. e.* in my hands it works to perfection, but not so in the hands of others. I have now dispensed with my assistant and all my old pluggers, and propose to use my spring plugger entirely. I have asked my patients who have had teeth filled with the mallet and plugger, which they liked best, and they have said "the plugger," because the force of the blows is more equal. A spring mallet, which you can use handily, will save you an immense deal of work. Dr. Ellis, of Chicago, has used it and said to me, "It has saved me one-third the time, and I know it has saved me three quarters the strength; all you have to do is to hold the instrument firmly on the block." [The Doctor exhibited his spring plugger to the members present.]

Dr. Cushing: I tried this instrument in four or five cavities, and found it to work admirably for a person who was not accustomed to use it. I think it would work particularly well in small crown cavities, where the pressure would be direct. I think very much time is saved by the use of it. When the instrument is perfected it will work very nicely. I oftentimes tried the mallet and this plugger on my patient, and inquired of them which they liked the best, and they gave their preference to the blow of the plugger over that of the wooden mallet, remarking that it was pleasanter on account of its uniformity.

Dr. McClellan: Filling teeth may be regarded as the capital operation of Dental Surgery, therefore, whatever is calculated to facilitate the operation, and make it easier to the patient, or economize the time of the operator, are essential points to which we should direct our attention.

One word in regard to annealing gold. I can say, from practical experience, that annealing it in a pan is better. I anneal my gold in a closed box, heating it by the blowpipe, emptying the gold when heated sufficiently to use it. The best substance from which to pick the gold up (and I have tried various, such as plush, velvet, etc.), is spunk. It presents a uniform surface and is of uniform elasticity. It is the best substance I have ever tried, to line the gold pan on the bottom.

A few words in regard to the preparation of cavities in teeth. You should have your plans well matured before commencing; examine the cavities and have the instruments selected, and not have a superfluous number of them. You should have in your mind the form of the cavity, and then go to work. I often separate and open cavities with chisels; I select the material for filling according to the case. If I deem that the cavity is difficult to keep dry, I select materials that will be least endangered by getting moist before the operation is completed. Most teeth I would not think of filling with anything except adhesive foil, although it might give me considerable trouble to keep the cavity dry. I think good plugs can be made of soft gold, but prefer No. 4 adhesive foil. On placing the plug in its position, with a foot instrument I mallet it firmly to its place; the cavity may be half filled in that way.

One of our members said he did not fill the fangs of teeth with Wood's Filling, though he used it for crown cavities. I think there are some crown cavities that it is next to impossible to use Wood's Filling in—cavities where a portion would have to go beneath the free edge of the gum. Such cavities I fill with tin foil plugs well malleted down, making the surface of Wood's Filling, which takes hold of it well. It has also the advantage of protecting the pulp. I regard Wood's Filling, if properly used, as second only to gold, I am sure a great many good operations are made with Wood's Filling; I have made a few myself. It requires no less skill to make a good filling of that than with gold. We should examine the walls of a cavity after filling it, and see that it is well filled.

There is another filling which I use, it is only temporary; yet some use it as a permanent filling. I refer to the filling known as "Artificial Dentine." I have seen fillings of that material of five years standing; it very much resembles Hill's Stopping. I have used it for five years, and think it is the best material that can be used in front teeth, where the walls are thin and frail.

Dr. SHADOAN: Before you sit down will you please tell me what a "foot instrument" is.

Dr. McCLELLAN: It is an instrument with the end shaped like a foot: I think there are two in Atkinson's collection.

Dr. Horton: I would ask of the gentleman, is artificial dentine anything like "Roberts' Filling."

Dr. McClellan: It is not. I would add that I have used the mallet for over a year, and have no assistant, as the operator knows what kind of a blow he wants, the best. You can not regulate the spring plugger; the blows are all of the same force.

Dr. H. A. Smith: As a non-conductor to be used in filling teeth I found spunk objectionable, because of the pain it frequently produced when first introduced into the cavity—will not attempt an explanation of this.

The best non-conductor for this purpose I have tried, is wood. It has the advantage also of stiffness or resistance to pressure in filling, when the lamina of bone is thin, covering the pulp. If the nerve is exposed, a false bottom can be made of it, by letting the edges of the cap of wood rest upon solid dentine, beyond the point of exposure. Thick Bristol card board can be used in the same manner.

I use wood that is tough and moderately hard. If cut across the fiber it conducts heat and cold less than when cut lengthwise or in the direction of the fiber. The bark of wood is a poorer conductor than the wood itself, and for the purpose mentioned might be used to advantage. The average conductibility of wood and tooth bone I think is relatively about the same. The hardest woods are not necessarily better conductors than the lighter kinds; but would select the softer class of woods as perhaps preferable in this particular. If I wish to render the wood more indestructible, I would simply saturate it with creosote.

Dr. McClellan: I would like to make some other remarks. I will endeavor to answer one question, that is, why spunk sometimes gives pain when placed in a tooth. I do not think it is from its conducting property so much as from its velvety surface gliding over the exposed dentine; cotton will produce the same effect. In all such teeth I wipe out the cavity with cotton moistened with creosote, before filling; I do not claim this as an original idea, at least so far as the preservative quality is concerned. I use it to destroy the sensitiveness of the dentine, so that the passing in of the gold does not excite the salivary glands so much.

Dr. H. A. Smith: Heat may be very slowly transmitted through light porous substances like asbestos or spunk; but if the mechanical relation of the particles of which it is composed are altered by pressure or otherwise, the motion of heat may pass rapidly through a substance which in its natural state was a poor conductor. It is the air contained between the fibers of asbestos and spunk which prevents the rapid transmission of heat through them.

If wood is placed in the bottom of a cavity, the pressure necessary to make a good filling over it could not thus alter its structure, and we are certain that its conductibility is the same under the filling that it was before being placed in the tooth. If we use the light porous substances usually recommended for this purpose, we are not sure but that they are by pressure made nearly as good conductors of heat and cold as some of the metals themselves used in filling teeth.

Dr. McClellan: If we have a tooth with a strong wall, perhaps wood would be as acceptable a material as any other. We are frequently called upon to fill teeth largely decayed, where the nerves are nearly exposed; in such cases I would prefer to use a conductor that would resist the action of the fluids of the mouth and the atmosphere. I think dentine would be good for that purpose.

I saw a tooth that had been filled for years, half filled with dentine and the remainder with amalgam; that tooth served a good purpose for years; when I saw it, the non-conductor was exposed. Had that been wood or some substance like that, it would have allowed moisture to get in.

Dr. Taft: I find we are all the time learning; I have learned many things this afternoon which, I presume, will be advantageous to me; such little things, for instance, as the annealing lamp which was exhibited to us. I think this little arrangement is good, because the gold does not come in contact with the flame of the lamp. I like the arrangement very much indeed; there is, perhaps, a little difficulty in picking it up from the metallic surface of the pan; but you can pick up gold from any surface, from a piece of glass for that matter, if the instrument is sharp; and your instruments always should be kept sharp. Some operators, Dr. Atkinson for instance, picks up gold with the pliers, putting it in the cavity and not touching it with the point of the instrument at all until he condenses. There is an objection to picking up gold with the point of the instrument, while it is hot, as it will soon take the temper out of the sharp point, and it becomes dull and spoiled, and soon rendered unfit for use. I regard this lamp as a very good thing, and shall avail myself of its use as soon as possible.

I am very glad to see that there is a disposition in the profession to use the mallet, for I regard it as a great improvement on other methods. Everybody ought to use the mallet; it is not one of those complicated methods of operating that will involve anybody in difficulty. It is probably the simplest method of filling; the operator has only to handle his instrument, and the stroke is given by an assistant, if he has one. Everybody should have an assistant; but if he can not, let him get an automatic plugger by all means. Most dentists, I fear, on trying this instrument, if they find it will not work well at first, as they wish, will throw it aside and refuse to use it; it is much better to have an assistant, I think, though these instruments can be very well used after a little practice. I have used one similar to Dr. Deans' for some time. If you have an assistant, all you need do is to watch the point of the instrument, letting the assistant give the blow. After one becomes accustomed to the use of the plugger they might use it very well; but I would urge the profession everywhere to adopt the use of the mallet. I find I can work ten hours a day with the use of the mallet, easier than four or five by the old method; used to tire in the muscles of the chest. I know operators who use a great deal of force—jar the whole body when filling; many dentists of that class boast of the number of teeth they can fill in a day. With the mallet you can fill with perfect ease, and it is much pleasanter for the patient. There is not one patient in twenty who will not prefer it to hand pressure. You can fill more rapidly with hand pressure, but not as well as with the mallet, if you would take care. It is saying a good deal, but I think I can fill better with the mallet than in any other manner, and I know that every one who has practiced the use of that instrument for any length of time will tell you the same thing; it is only those who have never made a test of the matter who say they can not fill as well with the mallet as in any other manner.

The President: Filling teeth should interest us more or at any rate as much as any other subject that comes within the scope of our discussions. With regard to filling, all acknowledge that gold stands preëminent, and that there is nothing equal to it. Some prefer Abbey's, another one, Kessing's; another, Dunlevy's; another, Watts'. I am not so utilitarian as to confine myself to any one kind of gold; I believe I have used all forms of gold that have been presented to the dentist. I have found in some cases soft foil was better than adhesive, and in others, *vice versa;* but of later years I have confined myself mostly to the use of adhesive foil; at present I prefer Watts' crystal gold; the manner of using it is to anneal it, passing it through a lamp, piece by piece. This little instrument [pointing to the annealing lamp] has removed a weight off my mind. I would like to suggest to Dr. Metcalf, however, that instead of furnishing us an iron bottom, he furnish us with one of mica or isingglass, which I have found better than iron or any metal. I used to anneal gold by laying it on a sheet of mica, and passing it through the flame. Finding it took more time to manipulate gold after it was annealed, I made the pellets beforehand, and put them on the mica sheet and heated them red hot. I do so now.

The assertion was made that it was as difficult to put in a good Wood's filling in cavities where the gum came in contact with the cavity, as it was to put in one of gold.

We can not be too careful of the manner in which we prepare our cavities for the insertion of gold; where we have proximal cavities we must be very careful of that little piece of gum that is between the teeth. Moisture coming in contact with gold, be it adhesive or soft, in my judgment, spoils it; and I will never finish a filling that has a particle of moisture about it. Where the gum comes up into the cavity of the tooth, or the cavity is below the free edge of the gum, too great care can not be taken to preserve it from moisture; we must resort to one of two methods, either insert a wedge of wood or some such material between the teeth, or, if the gum is spongy and soft, crowd down the gum until we get the edge of the cavity above the piece of wood that is inserted; if the gum is hard, cartilaginous in its texture, as we will frequently find it, and the pressure would be too great upon the patient, it would be best to run a delicate knife between the teeth and cut out the gum until we get below the cavity. If there is much hemorrhage apply sulphate of iron, and then insert the wood; or, if there is time enough, I send the patient away, telling him to call again in two or three days when the hemorrhage has ceased and the wood can be inserted. In all cases where I have a filling to put in between the teeth—a proximal cavity that runs within a line of the gum, I insert a thin piece of wood, crowding it in firmly; it checks any exudation of mucus, and if the cavity extends to the neck of the tooth, the instrument strikes upon the wood instead of the gum. With such a cavity, if I have crystal gold, I care not what shape the cavity is, if it be only so that it will retain the gold, I can make a good filling. I have sometimes worked in where the cavities run far up into the dentine, on the very root of the tooth.

As regards the mallet, I think it of great benefit, especially for young operators (I do not mean to cast any reflection on Dr. Taft). The young operator finds the greatest difficulty in making his filling secure; by the use of the mallet he can condense his gold in such a manner as to make it perfectly solid. The mallet is a good instrument, and fillings can be put in with it magnificently. I think also they can be put in well without it.

Dr. Smith has spoken of spunk producing pain; we will find other materials as well as that producing pain. I look upon the production of pain when spunk is introduced into a cavity, as due to its power of absorption. I usually dry out cavities with bibulous paper.

Let us give this subject all the consideration we can; let us investigate it to our utmost, so that we may become perfect masters of it; until we do so, we can not expect our operations to be those of first class dentists.

Dr. Taylor: I think the mallet is a good institution; I do not regret its introduction in the profession as I do many other things. I recommend young students to commence with using the mallet, not letting the mallet use them. I believe there are some cases where it can be used advantageously, and others again, where it can not facilitate or expedite an operation. I believe some operators can fill a tooth with the old method as good as it can be filled by any method. The best method for me to introduce gold into a tooth, is the one which I have thoroughly mastered. There are some cavities which can be filled better by the old method than by any other. One method may be good in one instance, while it may be bad in another. Take, for instance, a cavity in the lower molar, near the gum; say it will hold a sheet, or half a sheet of gold, and the mouth is full of saliva all the time. I take to finish the preparation of the cavity a flat drill, make the base of the cavity a level floor, the walls being perpendicular. I would roll a strip of gold, which would be adhesive (which I prefer almost always), I would roll the gold as hard as I could, put it into the cavity, and have two or three other strips convenient to put in after the first one. The cylinder introduced is a little longer than the cavity is deep; so soon as it is placed in the tooth it is condensed, when another cylinder is introduced. In this manner the tooth can be filled in one-half the time it could be by the mallet, and there is no chance of moisture getting at the tooth during the operation; all that is necessary after plugging, is to file off the end of the cylinder and burnish it down, and the operation is perfect. This is a class of fillings, probably, where it is very difficult to keep the tooth dry. There are some cavities where I use the mallet to great advantage. I always roll the cylinder of gold exactly to correspond with the cavity; after laying in one, two, and sometimes three pieces with the plugging forceps, I take the instrument and consolidate them down, expecting, by doing this, to prevent the moisture from getting in.

One word on dental materials. I think the profession is going backwards on that subject. I doubt whether we are much better off now than when we had only gold and tin foil. I think it is a great mistake that we have now any other material. I would cast aside every filling but gold and tin foil. In filling a tooth, instead of using first tin foil and then Wood's Filling, as my friend from Louisville (Dr. Shadoan) says he does, I would use tin foil entirely; it makes just as good a filling. I have seen tin foil fillings which have been in for thirty years. I place gold as the first material which we should all use, and tin foil next; and I would place amalgam a way down, so low, that you could never get at it. (Laughter.)

Dr. Berry: I agree most heartily with the remarks of Dr. Taylor in regard to tin foil. It is very nice to talk about the beauty of gold fillings—it is *the* material for filling teeth; but, suppose a poor servant girl comes into your office, can she afford to pay you from $3, to $5 or 6$, for a gold filling? or can you generally afford to put it in for what it will cost you, or less? In such cases tin foil is often a good substitute to fall back on. I saw last summer two teeth extracted by the dentist who filled them in this city twenty-two years previously with tin, which had preserved them as well as gold could have done.

The President: The Doctor seems to ask a question of the chair, viz.: How a dentist can fill a tooth for a poor person with gold, and charge the same price as if it were of baser metal? I have exercised the benevolence which the Doctor alluded to, and have found it paid me, though I did not get the full price for it at the time. If I did not get the pay from the patient, I was sure to get it from some one with whom she would come in contact and converse. I can find many mentioned in my register, whose custom I never should have got, but for filling a tooth with gold for a servant girl and charging her no more than if it was of baser metal.

Dr. Horton: I don't know but what the members of this association are like a chap I once heard of, who said, if any body could tell a story that could beat him he would like to see him—especially if the other man told his story first. (Laughter.) Some gentlemen have mentioned cases of teeth being filled with tin foil over twenty years. I exhibited last year, at Niagara Falls, a tooth which I extracted for a gentleman 86 years old, and which he said was filled by Dr. Alexander, of New York, in 1802; I extracted it last season; that would make the filling 62 years old; it was in good preservation, and tin foil had evidently been used for filling it.

I do not want to boast of my benevolence, but I will say that I have filled teeth for many who were not able to pay the full fee, and, in some instances, my benevolence has extended to filling teeth for poor people, with the best material and in the best possible manner that I could, and must say that I never lost by the operation.

I consider gold the best material for filling teeth. I usually use No. 5 or 6, very soft foil, rolling it into pellets, the size depending on the cavity to be filled. I prepare the gold to fit the cavity, unless I can not make a pellet large enough for the cavity, then I adopt Dr. Taylor's plan and form a nook or corner that will receive the soft foil first; I then prepare some small pieces of Abbey's No. 3 or 4 adhesive. I make preparations to prevent the tooth from being flooded by the saliva, by sometimes inserting a piece of bibulous paper over the mouths of the ducts.

Dr. Talbert: If the tooth was filled with gold, and came in contact with a base metal, there might be galvanic action.

Dr. Ulrey: This is an important question. I have had about twenty-seven years experience in filling teeth, and using gold. I have found that whenever a gold filling rested on a tin filling, there would be galvanic action produced by the two metals in the mouth; that is what I have always attributed it to. I avoid, if I can, putting two metals in one mouth.

I once attempted to use artificial bone filling. I never charged any one any thing for putting in a filling of that kind. The next material to gold is tin foil; I have not used Wood's Filling. I think good adhesive tin foil is nearly, if not quite, as good as gold.

The President: I was going to mention, just as Dr. Horton sat down, a similar case to the one just related—that of my father. He was out shooting geese; he had discharged his gun, and it gave him what is termed a "kick," when, having some shot in his mouth, he found that one of them had lodged in one of his teeth, which was decayed. A friend who was present tried to get it out, but did not succeed; it gave him a great deal of pain; by biting upon it, it stopped up the cavity, and answered all the purposes of an ordinary filling. So it remained till the time of his death.

Dr. Horton: A gentleman who was at the excavation of Pompeii said he found dental and surgical instruments that were better than some we have at the present day; they had been underground for nearly 1800 years; but the Italian government have lately refused to allow any thing to be brought away, so he did not get any of them.

Adjourned till 7 o'clock P. M.

### EVENING SESSION.

Subject — *Diseases of the Teeth; Pathological conditions; Sensitive Dentine; Alveolar Abscess; Absorption and reproduction of the Dental tissues.*

Dr. Watt: The subject of the reproduction of bone is not as extensively understood as it ought to be. I remember when Dr. Taylor wrote a paper some years ago, which was published in one of the Journals, in which he said that bone could be reproduced, he was looked upon as a willful liar. It is very well recognized that the reproduction of bone will take place whenever there is periosteum to guide it; a piece of the periosteum can be taken out and you can make it into any shape you please; periosteum is recognized as the ordinary bone producer; and I think when the war is over, and all the surgical operations have been brought to light, it will be well known that not only is periosteum the bone producer, but also that the system is capable of producing periosteum. If that is not so, why is it that you can take out forty-three lines of the length of the humerus, and have the bone reproduced? I take it for granted that when Driggs passed the probe through the palate bones three lines by fifteen, that there was destruction, not only of the bone, but of the periosteum. The bone was reproduced. How? Can not nature which can go to work and make bones, blood vessels, muscular tissue, skin, hair and nails, make periosteum? It takes a long time to understand that fact. Many, I suppose, have noticed a cicatrix on my forehead; I had a good deal of pleasure in watching that, and never had any thing pay me better in my life. (Laughter.)

I would sit before a lookingglass and watch the re-formation of the bone; the periosteum and the skin became developed, and my face is as you see it now. Bone is produced not directly by the periosteum, but by its influence. What is to be done for the reproduction of alveolar tissues? Simply give rest and freedom from irritation; eating various solids and drinking certain fluids may produce irritation, though the atmosphere is the greatest irritant to prevent the re-formation of the bony tissues; when you can keep a part at rest and exclude the atmosphere, in a reasonable constitution, you may expect the reproduction of bone, even if the periostoum is all gone. You will not always have a perfect formation, but you may expect reproduction of bone.

I think usually our treatment is too heroic. I think Dr. Driggs was far too heroic, and that he could have got along best otherwise. I think a solution of chlorine in water is usually stimulating enough in almost any case. It is a good disinfectant, and is usually heroic enough.

Sometimes something else is necessary, but if you *wish* to have new tissue created this is the best article to resort to. "Cleanliness," you know, "is godliness," and more especially so in these cases.

Remove the dead substance and exclude all irritants, the greatest irritant being the atmosphere. Acting on these general rules, I believe that almost any one can treat any case of the reproduction of tissue, whether bony or otherwise. Frequently it is well to apply a watery solution of glycerine around the tissue where you wish to build up. Also, apply the solution around the gums between the teeth, and there will be very little trouble from oxygen for the next twenty-four hours.

Dr. Taft: There seems to be very rapid progress making in this direction; I do not know in what particular in dentistry progress is being made so rapidly as in a knowledge of the pathological conditions of the teeth and the treatment. Especially is this true in regard to the subject of the reproduction of bone—reproduction of the tissues about the teeth, the treatment of diseased conditions of the teeth that were formerly supposed to be altogether beyond restoration. The dental profession is making advances in this direction (as Dr. Watt has intimated) perhaps more rapidly than any other. As the attention is now being directed particularly to this subject of the absorption and reproduction of bone. Physicians many years ago scouted at the idea of the reproduction of bone, but they have for the last few years awarded enconiums to the dental profession for their successful attempts in this direction. There are now so many well attested cases of this kind of restoration that it is no longer to be doubted. I have seen patients who were loathsome objects to behold, who were rotting down, if you will allow the expression, built up; new tissues were formed, the gums reproduced perfectly. It is not a year since I saw a case of this kind: It was that of a man who was so loathsome that he did not venture into society, whose breath would give an unendurable stench wherever he went. His front upper teeth were all gone and you could have put your three or four fingers where the anterior portion of the palate formerly was, up into the *nares*. That case was cured so that the tissues and mucous membrane looked as well as I have ever seen them. You can not cure in all cases. Beginners should take the favorable cases. The case I have mentioned was one Dr. Atkinson had, and I was perfectly astonished to see what proper treatment could do. I have several cases in my own hands where the teeth, loose from necrosis or waste of the alveolus, by proper treatment became solid. Dr. Pease mentioned some extraordinary cases before the American Dental Association last summer. These cases present encouragement, and we should go on and accomplish all that can be done in this direction. To treat these cases efficiently, it requires a knowledge not only of the parts affected, but of the whole system; and we must also understand the nature of the agents we employ. When we have studied the nature of these conditions, have a good knowledge of the anatomy and physiology of the parts affected and also studied the nature of the operation of the therapeutic agents and their influence upon living tissue, then we are ready to begin our work.

The treatment in these cases is very similar. The treatment of Drs. Watt, Driggs, Pease and Atkinson, as well as a number of others throughout the country is very much the same. They all agree that rest is necessary, that a quiet of the parts must be had; that you must exclude all irritation; that a plasm must be formed for the developement of new tissue, and, you can encourage by proper treatment that devolopement. These are only the landmarks as it were which will apply as well to one case as to another.

I have nothing to say on the rationale of the cure, as Dr. Watt has referred to the chief points.

This is a subject we ought *all* to study. A great many in the profession will refer to one, two, or three as being engaged in the treatment of these cases, snearingly, as if it was a mere pretense. Such assertions are made only for the purpose of shielding their own ignorance; they either do not know or do not care to know. Whenever a man refers to another performing this operation as a humbug, you may look upon him as either ignorant in regard to the subject or—something else.

Dr. Taylor: I suppose if any one twenty years ago had asserted that there could be reproduction of tissue he might have thanked his stars if he had not been driven out of society. It was about twenty years ago that a gentleman of our profession, residing in another city said, in my presence, hat he had cured alveolar abscess; I did not believe him then; and think still he had not then an idea of the nature of the disease; now, I would believe any of you gentlemen if you told me you had succeeded in curing alveolar abscess and restored the parts to health. The fact is, gentlemen, we have got to that point in dental progress where we will believe any assertion based upon correct physiological and pathological principles. As my friend Dr. Watt has said, if the system can restore bone, why not restore periosteum? If all the tissues of the body can be restored by the body itself, why can not the periosteum be restored? Take the case reported by Dr. Driggs, a report which I would recommend to all for its conciseness—take that case, it was one in which there was not only a reproduction of bone but a reproduction of periosteum. The question now is whether this bone is reproduced independent of the periosteum or not. In some cases we have no doubt at all that the periosteum reproduction is probably incident to that of the osseous structure; and we have no doubt that the bone itself throws out a plasm and ossification takes place. If we have a plastic condition of the system, that is, a condition that affords the most abundant material for the reproduction of tissue, we shall have no trouble; if the system is not abundant in the necessary elements, suppuration comes on.

Dr. Smith in his paper, mentioned the case of a fang being destroyed; the question would suggest itself, what becomes of the periosteum? and how can a sack be got at the point of the fang for the reception of the matter. These parts have to be healed first; there is not only reproduction of the periosteum, but, if there has been absorption of the bone, reproduction of it also.

If these cases are treated at all, they should be understood clearly. I have no doubt that alveolar abscess can be cured, and I think the profession are beginning to believe this.

Dr. H. A. Smith: It is now generally conceded that bone is reproduced after being taken away from the periosteum,

Certain conditions are necessary however, to render regeneration possible. The periosteum must be carefully preserved and of considerable thickness, the patient of a good constitution and observe strictly hygenic laws.

Even under the most favorable conditions it is doubtful if the osseous material included by the periosteum performs all the functions of the original bone in its integrity. Several cases have been mentioned in which the alveolar tissue has been reproduced about the necks of the teeth. It is possible to effect this if the periosteum of the tooth can be first reproduced. Then we may have an osseous matter resembling the bone deposited upon it. The reported successful results in this direction by a few in the profession, should certainly stimulate all to an investigation; particularly to what extent dental tissues may be reproduced.

Dr. Watt: I think I have stated that periosteum is the only bone producer; but I say that the God of nature has so organized the animal system that it can reproduce periosteum as well as other tissues. If Dr. Smith would see the operations made in the hospitals in the army he would believe this fact. The effort should always be made to get out all the diseased bone, then cover the other portion up and keep it well protected. I will give a case with which I was familiar:

A young man who belonged to the 12th Ohio Volunteer Infantry, went out in the three months service, and in a battle was wounded; several inches of the humerus was taken out; and that young man went back in the army, fought at the second battle of Bull Run, through an entire campaign, at South Mountain and Antietam, where he fell mortally wounded and died.

Dr. Baxter: What constitutional treatment is required to assist in the reproduction of bone?

Dr. Watt: That is a very important point. It is very unwise to make a surgical operation without regard to the system. It is well to have the system in the best possible state of living. Where there is defective nutrition, the digestion is tolerably good, a reasonable appetite, and yet there seems to be a defect of the general functions (such cases you will often find). In such cases I have used the phosphate of lime a great deal; in more delicate cases I have used a combination of the hypophosphates.

Dr. Horton: I was in the army hospital at Washington some time ago, where the surgeon showed me a dozen cases of exsections; one or two were in a condition to walk around while some had gone away cured. I would not have believed there were such cases, had I not seen them.

Dr. Taft: I think Dr. Smith is fighting an assertion which has never been made. It seems to me, as Dr. Watt has stated, that periosteal tissue may be produced from soft or hard tissue. I think the case of his forehead illustrates one great principle in connection with this subject.

Adjourned till 9 o'clock A. M. to-morrow.

The subject of yesterday afternoon was continued.

Dr. Ulrey: Although I have had a good deal of experience in sensitive dentine, I am not prepared to state what is the best method of allaying that sensitiveness. Our old methods were to use creosote and arsenius acid; but, so far as I know, these are pretty much abandoned. I occasionally use the chloride of zinc; two or three applications of that I find answer in most cases. If it is a cavity that requires a temporary filling, I find it best to use Hill's stopping, letting the patient keep the mouth well cleansed with alkali for some weeks or months, as the case may be. I then remove the temporary filling, and do the work without difficulty.

Dr. Taylor: Will chloride of zinc act upon the dentine.

Dr. Ulrey: Not in my experience. I am aware that it shrinks the fibers back, but I do not think it has any very bad effect. Where the pulp is exposed I do not use it. It acts well in cavities where the nerve is not exposed.

Dr. Baxter: I always pay attention to three points; first, to keep the cavity dry while operating; second, to use a sharp instrument; and third, as Dr. Atkinson would say, to "work boldly."

The President: The best agent that I have ever used is the fluid extract of aconite applied to the dentine by a little piece of cotton saturated with it. I think it is the best thing I ever used. I had a case of a young man where the dentine was exceedingly sensitive. I made an application of that for a minute or two and the sensation was that of pain at first; when the pain stopped I worked on the dentine without any difficulty whatever. I took off quite a cutting of bone before I came to any sensitiveness.

Dr. McClelland: I should like to get along without the use of escharotic agents, but there are some cases where we can not get along without their use. The objection to chloride of zinc is, that it creates considerable pain. I applied it to the tooth of a young man, and the sensitiveness lasted several hours. I usually use the ordinary application for destroying the nerve—a mixture of morphine, creosote and arsenic; a little of this is placed on a piece of cotton, put in the cavity and then some of Hill's stopping is applied. I do not think it is generally safe to apply it to children's teeth; it requires considerable judgment as to the amount to be applied and the manner of applying it. I made an application of it once in two very sensitive proximal cavities of a little girl eight years old, and found it to work very well.

Dr. Watt: A very good article on the subject of escharotics will be found in volume ten of the *Dental Register*—the first article.

### *Mechanical Appliances and New Inventions.*

Dr. McClelland exhibited to the Association his "universal coffer dam" to prevent saliva from going into the tooth; the "foil crimper," and also an appliance for regulating the teeth.

Dr. Watt: There are many cases, especially among young girls, where a lateral incisor will be seen protuding out from its place. In such a case I give them a small hickory rod, telling them to put one end of it on the tooth, the other on the table or desk at which they are studying, and keep pressing on it. I find the plan to work very well.

Dr. Taylor: I sometimes adopt that plan to throw out lateral incisors.

Dr. Keeley: I am reminded of a patient who once called on Dr. Taylor to have a lateral incisor brought to its proper position; he thought the doctor would give him some complicated appliance, but, to his great astonishment he picked up a little piece of wood and fitted it around the tooth, requesting him to wear it.

Dr. Taylor: We do not look enough oftentimes to see how nature will help us. A lady in this city had a very irregular set of teeth; she called on a dentist to have them regulated, and he said he would charge her $50. She could not afford to pay that and came to me. I told her to have one bicuspid removed, which she allowed me to do, I charging her fifty cents. In a year or two the teeth were perfectly symetrical.

Dr. Berry: I suppose we have all seen cases where teeth have been moved simply by a slight pressure of cotton. I have never failed when I used a stick, to move a tooth. I have treated several cases of young ladies in that manner; I find they are teaching each other now, so I loose considerable custom. (Laughter.)

Dr. Taylor: I want to ask the members what I will do in this case: a young lady has, by pressing her tongue against her upper teeth forced them out; I found I could not force them back again without their coming in contact with the lower teeth.

Dr. Berry: I would extract one of the lower teeth.

Dr. McClelland: I would make a cap for the upper teeth and a cap for the lower teeth, which would draw them upward and inward at the same time. [The Doctor illustrated the shape of the appliance.]

The PRESIDENT: Can any one tell me what to do in this case: the edge of the right central incisor of a young lady is turned inward. I have directed her to wear a band, which she has done constantly for the last eight months; while the band is on, the tooth is in the right position, but when taken off it comes back into its old shape.

DR. WATT: Make her eat phosphate of lime like bread and butter for about two weeks.

The PRESIDENT: Thank you; I will try that, Doctor. The young lady is very handsome, and I have been treating the case for the last two years; she is very anxious that the tooth should be brought back in its true position.

### *The True Position of the Dentist, in Relation to his Patient, his Student, and his Profession.*

DR. KEELEY: I think we do not advise our patients enough in reference to taking care of the teeth; we should try, I think, on all occasions to impress them with the importance of keeping their teeth in proper condition. Sometimes by simply taking one tooth out we can regulate a whole set of teeth. A great many dentists will take a wrong tooth out just from the fact that they have not studied their case. How often is it the case that just by a little advice, which may not take us over five minutes to give, we can do our patients immense good. Who of us on seeing some of the teeth standing very irregular, do not regret that we had not seen the case sooner so that we might have extracted a tooth. We can oftentimes avoid making appliances if we advise at the right time. I never feel better satisfied with myself than after giving two minutes advice to a young patient, when I know they will follow it, and afterward thank me for it.

DR. SHADOAN: I was not in during a part of the discussion on irregularities of the teeth, and would like to ask the Association in regard to the case of a young lady sixteen years of age. The temporary tooth is still in the mouth and the permanent one is coming out; the temporary tooth is quite as large as the corresponding permanent one on the opposite side. I should like to know whether it would be better to take out the permanent tooth and let the temporary one remain, or not.

DR. BERRY: If you took out the permanent tooth the temporary one would last ten or fifteen years, when it would come out. It is not unusual for a temporary cuspidati to remain till the person is from twenty-five to thirty-five years of age.

DR. SHADOAN: I told the young lady it was a peculiar case and I could not be prepared to decide upon it till I came back. I have a sister-in-law thirty-five years of age who has a temporary tooth still, and the permanent tooth has not yet made its appearance.

DR. TAYLOR: One word on what our duty is to the patient and student. I regard our duty to the patient to be such that it not only requires us to give him the best of our skill in manipulating, but it is also our duty, as Dr. Keeley remarked, to give him the very best advice we can. It is to our interest to preserve all the teeth we can for his use, and to prevent decay; it is not only our interest to do so, but our *duty*, and whenever we can give any such advice we should do it.

A few words in relation to students. I think the time has come when we should give them that advice which will be for their good. Take no student unless you know he will pursue a proper course, such as will make him a good dentist; take no student whose preparatory course you think has been insufficient. It has been a great drawback in our profession, this taking students whose preparatory course has been inadequate. There are some dentists that take students who can not speak their own language intelligibly, or write a sentence correctly. These unprepared students can not take hold of a study with that clearness which one that has been prepared can do. Besides all this, we have a literature, in our profession, to sustain. We all feel this, especially those of us who have been in the profession for twenty or thirty years, watching its progress. Twenty years ago we could never have got so many members of our profession in one body as there are here to-day and discuss such subjects as we have done—such as the pathological conditions of the teeth; we can now talk with the physicians on such subjects. Let us see to it that all our students have the requisite requirements.

Dr. Taft: This is a very important subject, and one so far as the future is concerned, has no equal. It is a very easy matter for us, who are here, to say that we will only have one class of students and no other; it is an easy matter for us to say that we will take no students for less than two or three years; it is not a difficult matter to say that we will not take a student unless he is prepared, as far as literary and scientific acquirements are concerned; it is very easy to say all that, but there is a difficulty that I hardly see how we can reach and overcome, viz.: there are so many in the profession throughout the county who do not and will not attend the meetings of our associations, who do not take the journals, and whom you do not reach at all. You will find them all over the country—in this city, in Louisville, and in every other city; they receive students for two months, perhaps, or for any length of time, so long as they get a fee, and send them out with a certificate, or recommendation of qualification for practice. How are we to remedy this? Our Louisville friends have mentioned that there are such men in their city, and we know there are some of this class in this city. Such students swarm over the country and bring the profession into disrepute. In many cases they bring down the scorn and the hate of the people upon them, and it takes in many instances, a long time to regenerate such communities. They are, to be sure, merely "quacks," and the people who employ them often have little or no regard for them.

We see some strange things in regard to this class of men.

I have a letter, which was handed to me recently, which came from a man who is practicing in Illinois, he is doing a flourishing business on the quack principle. He evidently does not know the first principles of the profession. (For the letter, see the February number of *Register.*)

That is only a specimen of the literature of this class of dentists. How would you like to present such a man as a "professional brother" to any scientific man or body of men?

This presents a lamentable feature in our profession, and if any method can be devised to remedy it, I shall be greatly rejoiced. The number of this class of dentists is, however, relatively if not absolutely diminishing.

What inducement is there for a student to pursue the best course and make himself thoroughly competent in every department of dentistry? Much every way; it redounds to his credit, it brings him honor, it brings him reputation, it brings him money, it brings him everything that is desirable; whereas, if he starts out in his professional course half prepared he receives none of these benefits.

These things ought to be presented in a tangible shape to the members of the profession whom we can in any way reach. Who are the men that are the mere dependants upon the profession, who present but a beggardly aspect in any sense? They are the men without attainments. This class of men do not recognize the responsibility that rests on them; if you converse with them you will perceive that at once. What can be done to remedy this state of things? Oh, that there could be a concurrent and systematic effort by the whole profession bearing upon this subject.

Dr. Berry: I want to say a word on this point here. This is a pretty difficult thing to remedy. I apprehend it requires a good deal of time and considerable effort on the part of our profession. I think the quacks get along quite as well in the city as any where else; but they may be found in all parts of the country. I suppose the only feasable mode of remedying the evil is to educate the people. I do not like the distribution of pamphlets, the main drift of which is to convince the dear people that their authors are the only dentists in their region proper to be entrusted with the care of the teeth.

I suppose the best method is to circulate tracts similar to the second edition of the *Dental Luminary*, published by the Cincinnati Dental Association, containing the simple truths respecting the management of the teeth which every one ought to know.

Dr. Watt: I think that we are too sensitive on some things and not sensitive enough on others. It is very evident that the better class of dentists, and the more sensitive class have been too cautious about enlightening the public; they were afraid it would verge on quackery; some of them got so far as to think it against etiquette to have a sign out, expecting they would be well known enough without one. That is all folly. It is the very highest style of egotism; it says "these other dentists have not earned a reputation as I have; people know me without my having a sign." Imagine a lady walking up and down the street, inquiring of a boot black, a street sweeper, or a soap fat man where you live! Our duty is not only to make our office comfortable and pleasant, but see that it can be easily found.

We have been far too dilatory in educating the public; this remark applies as well to country as city dentists. I do not agree with Dr. Taft that quacks succeed better in the country. It is a remarkable fact, Mr. President, that four persons, from the place of my nativity, without any preliminary study, without any extraordinary genius for the practice of our profession—that four such persons should be, under those circumstances, practicing in this city, and yet they are; but it is necessary for such persons to go away from home. I suppose if a man learns his quackery in the city, he will succeed best in practicing in a country town; and if he comes from a country town he succeeds best in the city.

Dr. H. A. Smith: Reference was made to the subject of dental signs. This matter seems to be regulated somewhat by custom and locality, and whether a sign should be eight by ten in *feet* or *inches*, I do not pretend to decide. Though I think the dentist who chose these proportions in feet exhibits decidedly bad taste.

The custom of adorning dental signs with the title of *M. D.* I think should be deprecated. If the dental profession is to take the high rank among the professions that we think it justly deserves, it certainly must be effected through its own merits. Those of the profession who have had the degree of Doctor of Dental Surgery conferred upon them, can hardly be excused for doing that which can not but bring the science of dentistry into disrepute. It may be that these M. D. dentists are a superior order of practitioners. If so, I must confess that I had failed to discover it, and believe the "generous public" have been equally slow in making the discovery. There is a strange inconsistency in the man who could talk so eloquently in our society meetings of the great value and importance of *dental* education, and at the same time have upon his door plate "John Smith, M. D., Dentist." Some even who are educators and who each year are responsible for having conferred the degree of D. D. S. upon one or more unsuspecting young man who desired to enter the dental profession, are in this public manner repudiating that which those graduates are led to believe is a guarranty of the confidence of the public. I would not be understood as under valuing the importance of medical in connection with dental education. I am induced to refer to this subject by an earnest desire that we should as a profession receive the respect certainly due us from an intelligent public, but which I fear is now partially withheld, and for the reason chiefly that we do not quite enough respect ourselves in a professional sense.

Dr. Berry: I do not think it strange, Mr. President, that students are often neglected by their preceptors; they are usually troublesome to manage when put at hard study as they should be, and the fee commonly trifling as compensation for instruction in the principles and practice of dental surgery. I do not think there is a dentist in good practice who can afford properly to instruct a student for less than one thousand dollars, and then he would be poorly remunerated if faithful in discharging his duties to his pupil.

Dr. Taylor: One of the duties of the profession is to see that we have a class of educated dentists. This is a duty that every one of the gentlemen present to-day, owe to themselves and the profession. Some of you are in duty bound to take students; you should do it and you can not discharge your duty to the profession unless you do so. Suppose we should all say we would not take students, what would be the result? Suppose we should leave all these young men to be taken care of by our Dental Colleges? In a little tour which I made some time ago I met twenty students and not one of them had been in any institution. If all our dentists would send us students we would have fifty in this college every year. There are men present who have been in the profession ten or twelve years who have never sent a student to any college. I will talk plain because I think it is my duty—a duty which I owe to my profession and to myself.

A large majority of us have not done our duty as we should. It is our duty to send students to our institutions so that they may be prepared to discharge the duties that devolve upon them as dentists.

A word in relation to this preliminary education which Dr. Smith spoke about. The idea of sending students to college is to let them have this preliminary education. If you took a student to-morrow, and it was the last day of October, you should send him to the college three months where he would get in the traces, while otherwise, it might take three years.

Dr. Berry: In order to remove this evil of incompetency in the dental profession, we should be sure that our students understand everything as they go along, and require them to attend two courses of lectures at a dental college.

Dr. McCollum: I feel that Dr. Taylor's remarks had some reference to me. I have not sent a student to the college for a number of years. I have had several students whom I have recommended to attend Dental Colleges as they could obtain a better knowledge of dentistry there than elsewhere.

Dr. Watt: I feel that Dr. Taylor's remark hit me pretty hard, and I will make a little apology. Some of my students have been graduates of this institution, but my latest student was voted out (Dr. Taylor voting against his admission) and so I do not feel like bringing any more. [Laughter.]

The discussion here closed.

The President said:

Gentlemen of the Association: We have met and had a very pleasant meeting. It has been, probably, of benefit to us all. Some who are here have not taken a very active part in the discussions. May we not ask them that at the next meeting they will do so. May we not, each one of us, during the three hundred and sixty-five days which will intervene between this and the next meeting, find something in our practice to present to the Association. The Bible says "Freely ye have received, freely give." Let that be our motto for the future year, and let us see if we can not have, in the meeting of another year, far more interest than was manifested in this.

The President then announced that the Twenty-first Annual Meeting of the Mississippi Valley Dental Association was adjourned till the second Wednesday in February, 1866, at ten o'clock, A. M.

